# Reproductive History of a Woman With 8p and 18p Genetic Imbalance and Minor Phenotypic Abnormalities

**DOI:** 10.3389/fgene.2019.01164

**Published:** 2019-11-20

**Authors:** Anna A. Pendina, Yulia V. Shilenkova, Olga E. Talantova, Olga A. Efimova, Olga G. Chiryaeva, Olga V. Malysheva, Vera S. Dudkina, Lubov' I. Petrova, Elena A. Serebryakova, Elena S. Shabanova, Irina D. Mekina, Evgeniia M. Komarova, Alla S. Koltsova, Andrei V. Tikhonov, Tatyana G. Tral, Gulrukhsor Kh. Tolibova, Natalia S. Osinovskaya, Mikhail I. Krapivin, Anastasiia V. Petrovskaia-Kaminskaia, Taisia S. Korchak, Tatyana E. Ivashchenko, Oleg S. Glotov, Olga V. Romanova, Anton E. Shikov, Stanislav P. Urazov, Viktoriya V. Tsay, Yurii A. Eismont, Sergei G. Scherbak, Yanina M. Sagurova, Elena S. Vashukova, Polina Y. Kozyulina, Natalya M. Dvoynova, Andrey S. Glotov, Vladislav S. Baranov, Alexander M. Gzgzyan, Igor Yu. Kogan

**Affiliations:** ^1^D. O. Ott Research Institute of Obstetrics, Gynecology and Reproductology, St. Petersburg, Russia; ^2^St. Petersburg State University, St. Petersburg, Russia; ^3^St. Petersburg State Pediatric Medical University, St. Petersburg, Russia; ^4^City Hospital №40, St. Petersburg, Russia; ^5^NIPT LLC, St. Petersburg, Russia

**Keywords:** 8p deletion, 18p deletion, 8p duplication, genotype–phenotype correlation, miscarriage, PGT-SR, prenatal karyotyping, NIPT

## Abstract

We report on the phenotype and the reproductive history of an adult female patient with an unbalanced karyotype: 8p23 and 18p11.3 terminal deletions and 8p22 duplication. The indication for karyotyping of the 28-year-old patient was a structural rearrangement in her miscarriage specimen: 45,ХХ,der(8;18)t(8;18)(p23;p11.3). Unexpectedly, the patient had the same karyotype with only one normal chromosome 8, one normal chromosome 18, and a derivative chromosome, which was a product of chromosomes 8 and 18 fusion with loss of their short arm terminal regions. Fluorescence *in situ* hybridization revealed that derivative chromosome was a pseudodicentric with an active centromere of chromosome 8. Array comparative genomic hybridization confirmed 8p and 18p terminal deletions and additionally revealed 8p22 duplication with a total of 43 OMIM annotated genes being affected by the rearrangement. The patient had minor facial and cranial dysmorphia and no pronounced physical or mental abnormalities. She was socially normal, had higher education and had been married since the age of 26 years. Considering genetic counseling, the patient had decided to conceive the next pregnancy through *in vitro* fertilization (IVF) with preimplantation genetic testing for structural chromosomal aberrations (PGT-SR). She underwent four IVF/PGT-SR cycles with a total of 25 oocytes obtained and a total of 10 embryos analyzed. Only one embryo was balanced regarding chromosomes 8 and 18, while the others were unbalanced and demonstrated different combinations of the normal chromosomes 8 and 18 and the derivative chromosome. The balanced embryo was transferred, but the pregnancy was not registered. After four unsuccessful IVF/PGT-SR cycles, the patient conceived naturally. Non-invasive prenatal testing showed additional chromosome 18. The prenatal cytogenetic analysis of chorionic villi revealed an abnormal karyotype: 46,ХХ,der(8;18)t(8;18)(p23;p11.3)mat,+18. The pregnancy was terminated for medical reasons. The patient has a strong intention to conceive a karyotypically normal fetus. However, genetic counseling regarding this issue is highly challenging. Taking into account a very low chance of balanced gametes, emotional stress caused by numerous unsuccessful attempts to conceive a balanced embryo and increasing age of the patient, an IVF cycle with a donor oocyte should probably be considered.

## BackgrounD

Terminal deletions are among frequent karyotype abnormalities in the adult population. Deletions of the terminal regions have been described for every human chromosome. The genetic imbalance caused by terminal deletions is considered to be a major source of multiple congenital anomalies and mental retardation (Gardner et al., 2011). Telomeric deficiencies cause a number of syndromes including 1p36 deletion (1p-), Wolf-Hirschhorn (4p-), Cri-du-chat (5p-), Miller-Dieker (17p-), 18q-, and 22q- (Schinzel, 2001). In many cases, apparently simple terminal deletions are accompanied with submicroscopic duplications, triplications, and inversions (Ballif et al., 2003; Ballif et al., 2007; Rossi et al., 2008). Patients with such a genetic imbalance rarely have reproductive attempts; genetic counseling regarding the way of conception and adequate approaches for genetic diagnostics of their embryos is challenging.

Here, we report on the phenotype and the reproductive history of a female patient with 8p23 and 18p11.3 terminal deletions and a 8p22 duplication.

## Case Presentation

The patient is 28-year-old woman who was referred to D.O. Ott Research Institute of Obstetrics, Gynecology and Reproductology (St. Petersburg, Russia) for cytogenetic analysis of a miscarriage specimen. The pregnancy was lost at 5/6 weeks of gestation. Conventional karyotyping revealed an abnormal karyotype in chorion with a total of 45 chromosomes, only one normal chromosome 8, one normal chromosome 18, and a derivative chromosome that resulted from a translocation of the chromosomes 8 and 18 with a loss of their short arm terminal regions: 45,ХХ,der(8;18)t(8;18)(p23;p11.3). The patient and her spouse underwent karyotyping which revealed a normal male karyotype in the spouse (46,XY) and an unbalanced female karyotype in the patient—45,XX,der(8;18)t(8;18)(p23;p11.3), the same as the one detected in the miscarriage specimen. The patient's parents were karyotypically normal.

The following genetic counseling revealed the patient's unremarkable family history. The patient was born after the second uneventful pregnancy (following the first ectopic pregnancy) of non-consanguineous parents of Russian ethnicity—a 29-years-old mother and a 34-year-old father. During the preconception period and the first trimester, the parents lived in Luanda, Angola—a territory with an elevated level of radiation because of uranium mines. The patient's mother worked as a translator; the father, as a radio operator on a MI-8 helicopter. In the second trimester, the parents returned to Russia. The patient was born in term by spontaneous vaginal delivery complicated by footling presentation and fetal asphyxia. Her birth weight was 3,200 g, her length—49 cm.

During the first year of life, the patient was followed up by a neurologist because of hypotonia and received vitamin therapy and therapeutic massage sessions. During childhood and adolescence, the patient was followed up by an endocrinologist because of obesity. The patient never demonstrated either behavioral or mental health problems. She completed a general primary school, received general secondary education and higher education. The patient works as a child psychologist. The patient had been married since the age of 26 years. Taken together, these data strongly indicate her good social adaptation.

**Figure 1:**
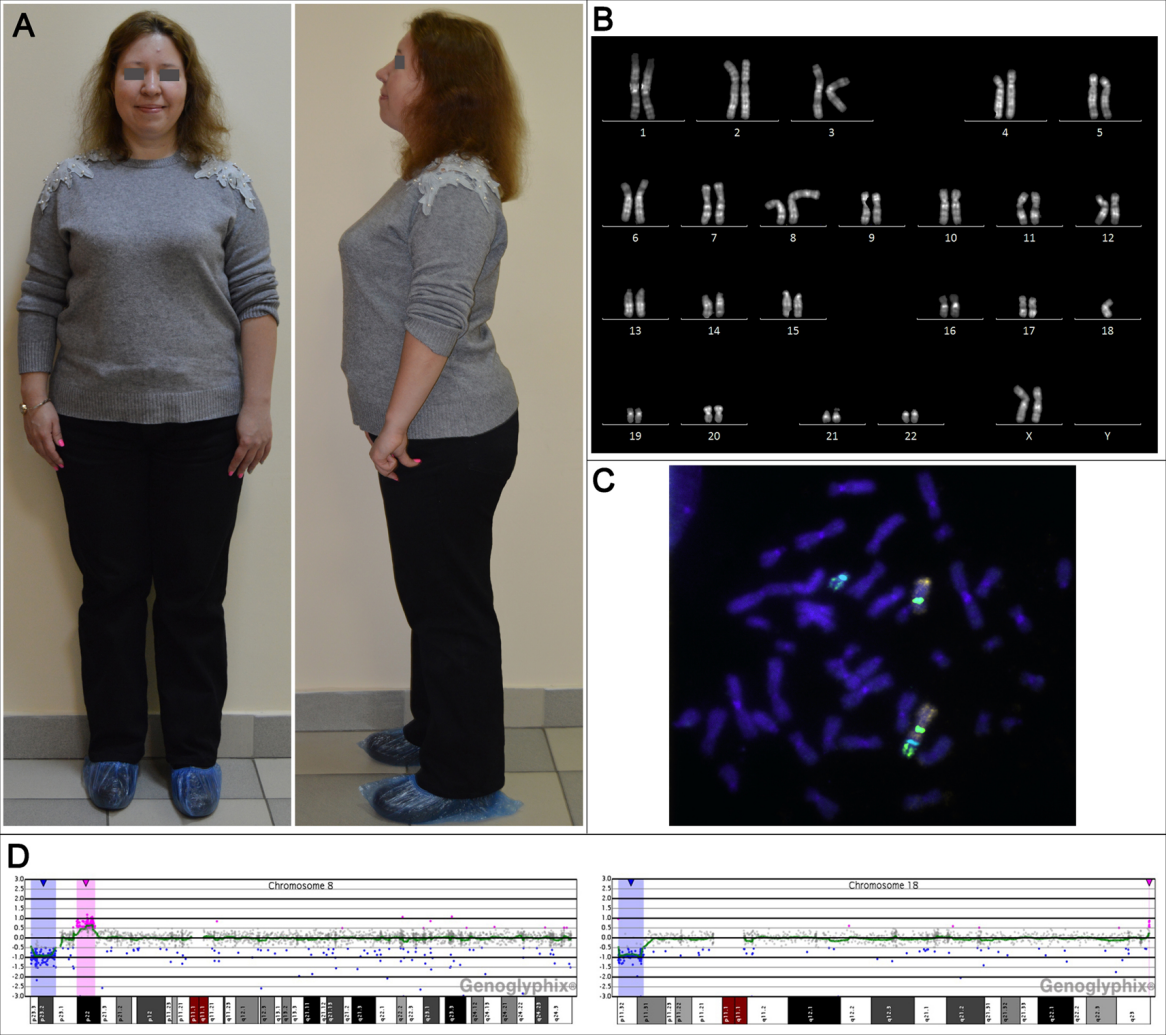
The phenotypic and genotypic characteristics of the adult female patient with unbalanced karyotype 45,XX,der(8;18)t(8;18)(p23;p11.3). **(A)** The patient's appearance at the age of 31 years. **(B)** The karyogram of QFH/AcD stained chromosomes from the patient's PHA-stimulated lymphocyte. **(C)** Metaphase chromosomes from the patient's PHA-stimulated lymphocyte after fluorescence *in situ* hybridization with DNA probes specific to chromosomes 8 and 18: Vysis CEP 8 (D8Z2) (green), CEP 18 (aqua) (Abbott Molecular), whole chromosome 8 painting probe (orange), whole chromosome 18 painting probe (green) (Applied Spectral Imaging). **(D)** aCGH results for chromosomes 8 and 18; deleted regions are marked with blue, duplicated regions are marked with red.

At the genetic counseling, the patient had first-degree obesity: her height was 158 cm (the mother's height—160 cm, the father's height—168 cm), weight—78 kg, BMI 31.24 kg/m^2^. The patient had minor facial and cranial dysmorphia: flattened superciliary arches, a narrow palpebral fissure, hanging eyelids, a wide nasal arch and a fleshy nose tip. She had a bit shortened arm length, a slight valgus deformity, and disproportionately large feet (**Figure 1A**). In general, the patient looks like her mother.

The patient had menarche at the age of 11 years, regular menstrual cycles of 28 days with menses lasting 4 to 5 days. By the time of the genetic counseling, the patient had experienced two naturally conceived pregnancies; both ended in a miscarriage at 5/6 weeks of gestation. Cytogenetic analysis was performed only for the second miscarriage specimen and revealed the above-described karyotype. An ultrasound examination of the patient's pelvic organs showed an unremarkable uterus and ovaries of normal size with 11–13 antral follicles. Her hormonal status was normal: follicle-stimulating hormone (FSH)—7.43 mIU/ml, luteinizing hormone (LH)—1.8 IU/l, anti-müllerian hormone (AMH)—1.31 ng/ml, prolactin—281 ng/ml, dehydroepiandrosterone (DHEA)—7.0 ng/ml, 17-ОНР—4.6 ng/ml, thyroid-stimulating hormone (TSH)—1.32 mU/l. Genetic testing for common *CYP21A2* genetic variants revealed heterozygosity for V281L substitution; the patient's spouse had no *CYP21A2* genetic variants. Considering the genetic counseling, the patient had decided to conceive the next pregnancy through *in vitro* fertilization (IVF) with preimplantation genetic testing for structural chromosomal aberrations (PGT-SR).

During the subsequent 2-year period, the patient underwent four IVF/PGT-SR cycles. In one cycle, an embryo transfer was performed; the pregnancy was not registered. After another two months, the patient conceived naturally. At early stages of gestation, the patient had an embryonic delay and a threatened miscarriage and was successfully treated with progestogens. At a gestational age of 9/10 weeks, the patient was recommended chorionic villus sampling for prenatal karyotyping. In spite of the known increased risk of having a karyotypically abnormal fetus, she refused to undergo invasive prenatal diagnosis due to the fear of pregnancy loss. As an alternative, she was recommended non-invasive prenatal testing (NIPT). The NIPT showed an additional copy of chromosome 18 material. An ultrasound examination of the fetus at 12/13 weeks of gestation showed total edema and an increased nuchal translucency (5.2 mm). The patient was repeatedly recommended prenatal karyotyping, and chorionic villus sampling was performed. The cytogenetic analysis revealed an abnormal karyotype with a maternally inherited derivative chromosome and an additional chromosome 18: 46,ХХ,der(8;18)t(8;18)(p23;p11.3)mat,+18. The patient was recommended to terminate the pregnancy for medical reasons. Dilation and curettage was performed. A subsequent histological and immunohistochemical analysis of the obtained endometrium showed no hormonal pathology: the expression of ER and PR receptors was in accordance with the gestational age. Four months after the curettage, the patient had normal results of the pelvic organs ultrasound examination with 10 antral follicles in ovaries and normal endometrial thickness.

## Laboratory Investigations and Diagnostic Tests

### Genetic Characterization of the Patient

Conventional karyotyping was performed on PHA-stimulated patient's peripheral blood lymphocytes. Preparation of metaphase chromosomes from fixed cell suspension and QFH/AcD staining was performed according to the standard technique with minor modifications described previously (Grigorian et al., 2010). The patient's karyotype was unbalanced, with a total of 45 chromosomes. There was only one normal chromosome 8 and only one normal chromosome 18. The aberrant chromosome was a product of chromosomes 8 and 18 fusion with apparent loss of their short arm terminal regions (**Figure 1B**). The karyotype was designated 45,ХХ,der(8;18)t(8;18)(8qter→8p23::18p11.3→18qter)dn.

To investigate the structure of the aberrant chromosome, fluorescence *in situ* hybridization (FISH) was performed on metaphase preparations using DNA probes specific to chromosomes 8 and 18: TelVysion 8p, TelVysion 18p, Vysis CEP 8 (D8Z2), CEP 18 (Abbott Molecular), whole chromosome 8 painting probe, and whole chromosome 18 painting probe (Applied Spectral Imaging). FISH signals were analyzed using a Leica DM 2500 microscope with a Leica DFC345 FX camera and the Leica Application SuiteV.3.8.0 software. The aberrant chromosome consisted of chromosome 8 and 18 material and contained FISH signals of both centromeres. Morphologically, only the centromere of chromosome 8 formed a constriction, indicating that the aberrant chromosome was a pseudodicentric with one active centromere—that of chromosome 8 (**Figure 1C**). The aberrant chromosome lacked both 8p and 18p subtelomeric regions. The breakpoints were in 8p23 and 18p11.3. Thus, conventional karyotyping and FISH showed a double partial monosomy involving 8p and 18p subtelomeric regions. The resulting karyotype was designated 45,ХХ,psu dic(8;18)(8qter→8p23::18p11.3→18qter)dn.

To determine the precise size of the deletions, array comparative genomic hybridization (aCGH) was performed (CGXv1.1 8x60K, PerkinElmer) using the protocol recommended by the manufacturer. DNA was extracted from peripheral blood lymphocytes. aCGH revealed a 6.718 Mb deletion in 8p23.1–p23.3, a 3.693 Mb deletion in 18p11.31–p11.32 and, in addition to karyotyping and FISH results, a 4.937 Mb duplication in 8p22 and a 0.060 Mb duplication in 18q23 classified as a copy number variation (CNV) (**Figure 1D**). Thus, the results of aCGH were as follows:

1. arr[GRCh37] 8p23.1p23.3(202133_6920415)x1
2. arr[GRCh37] 8p22(12582909_17519858)x3
3. arr[GRCh37] 18p11.31p11.32(146484_3839773)x1
4. arr[GRCh37] 18q23(77954106_78013620)x3.

The affected chromosome regions contained 44 OMIM annotated genes: 16 genes in 8p deleted region, 12 genes in 8p duplicated region, 15 genes in 18p deleted region, and 1 gene in 18q duplicated region.

### Outcomes of *In Vitro* Fertilization Protocols

The patient underwent four standard GnRH antagonist protocols. The outcomes of the protocols are summarized in the **Table 1**. In total, ultrasound monitoring showed 32 preovulatory follicles. A total of 25 cumulus–oocyte complexes were obtained by transvaginal aspiration. Of them, only 13 reached MII, 3 reached MI and the remaining ones degraded or were at the germinal vesicle (GV) stage. Intracytoplasmic sperm injection (ICSI) was performed for 16 oocytes (13 at MII and 3 at MI stage). After 20 h, two pronuclei were registered in 12 oocytes, three pronuclei—in 1 oocyte, and no pronuclei—in 3 oocytes. The embryos were cultured for 4–6 days under standard conditions. Since the third day of development, the embryos showed developmental delay, fragmentation, insufficient or absent compaction, and impaired blastulation.

**Table 1:** The outcomes of four IVF/PGT-SR cycles undergone by the female patient with unbalanced karyotype 45,XX,der(8;18)t(8;18)(p23;p11.3).

IVF/ICSI cycle	Treatment protocol	Follicles expected, n	Cumulus-oocyte complex, n	MII oocytes	Biopsied embryos on 3 day, n	Genetically balanced embryos regarding chromosomes 8 and 18, n	Outcome
1	GnRH antagonist + corifollitrophin alfa/HMG	11	4	1	0	–	No embryo transfer
2	GnRH antagonist + rFSH	9	9	4	4	1	Transfer of one embryo; no pregnancy
3	GnRH antagonist+ corifollitrophin alfa/HMG	8	8	5	4	0	No embryo transfer
4	GnRH antagonist + rFSH	4	4	3	2	0	No embryo transfer

**Figure 2:**
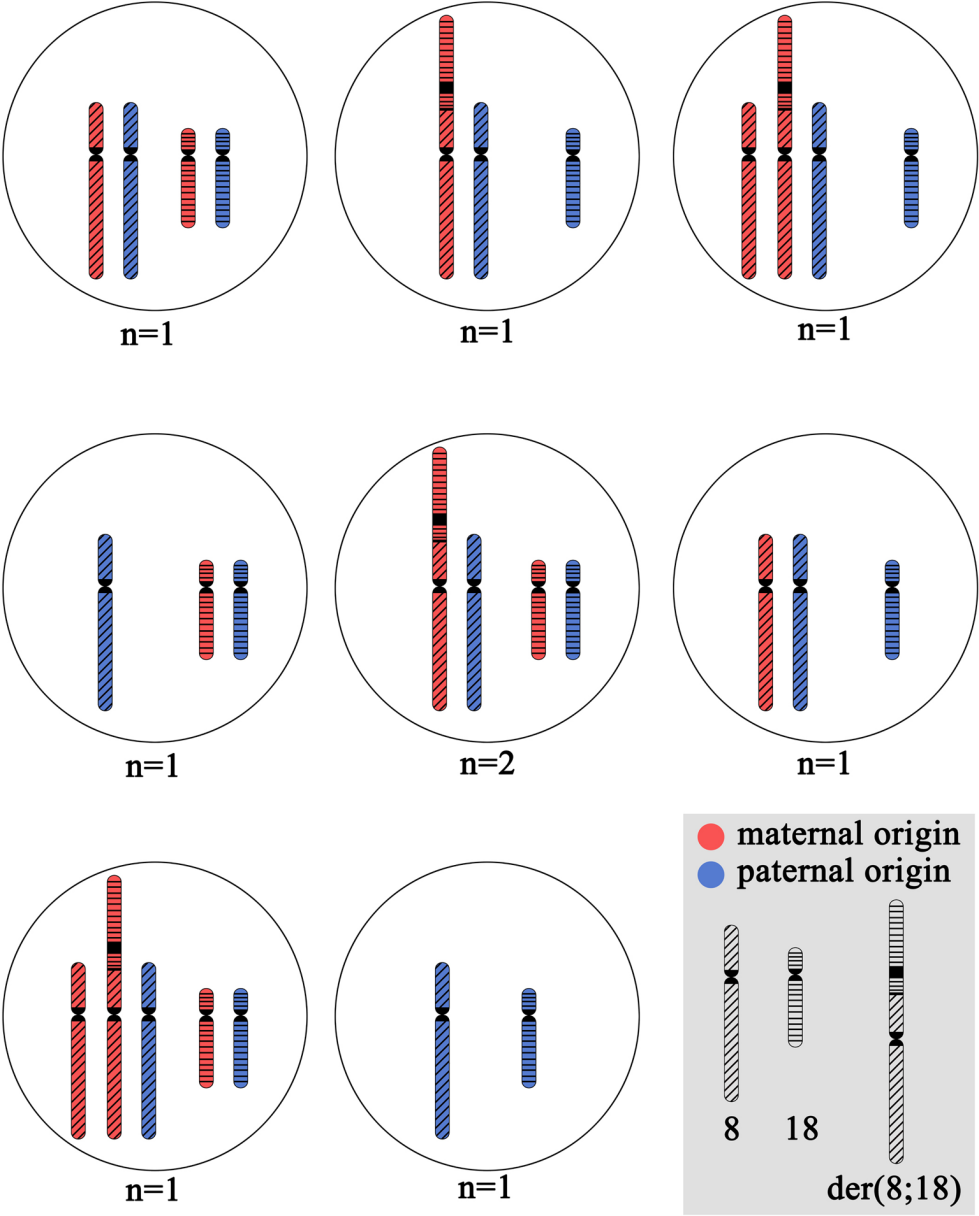
The cytogenetic picture of preimplantation embryos from the patient with unbalanced karyotype 45,XX,der(8;18)t(8;18)(p23;p11.3). The shown chromosome combinations are based on the revealed FISH signal patterns during PGT-SR with DNA probes specific to p and q subtelomeric regions and centromeric regions of chromosomes 8 and 18: TelVysion 8p, TelVysion 18p, TelVysion 8q, TelVysion 18q, Vysis CEP 8 (D8Z2), CEP 18 (Abbott Molecular). In all cases, the inheritance of normal chromosome 8 and normal chromosome 18 from the karyotypically normal farther is assumed. N indicates the number of embryos with detected variant of chromosome combination.

Blastomere biopsy was performed on day 3 for 10 embryos. PGT-SR was performed by FISH with DNA probes specific to the p and q subtelomeric regions and the centromeric regions of chromosomes 8 and 18: TelVysion 8p, TelVysion 18p, TelVysion 8q, TelVysion 18q, Vysis CEP 8 (D8Z2), CEP 18 (Abbott Molecular). In 9 out of 10 cases, the PGT-SR results were informative. In all but one case, the embryos were genetically unbalanced. The most probable chromosome combinations based on the revealed FISH signal patterns are shown on **Figure 2**. A total of seven genetic imbalance variants were registered among eight abnormal embryos. This advocates for a variety of chromosome disjunction patterns with no obvious prevalence of either variant (**Figure 2**). The only genetically balanced embryo developed to a 3СС blastocyst (Gardner grade) by day 6 and was transferred. The pregnancy was not registered.

### Outcomes of the Patient's Naturally Conceived Pregnancies

**Figure 3:**
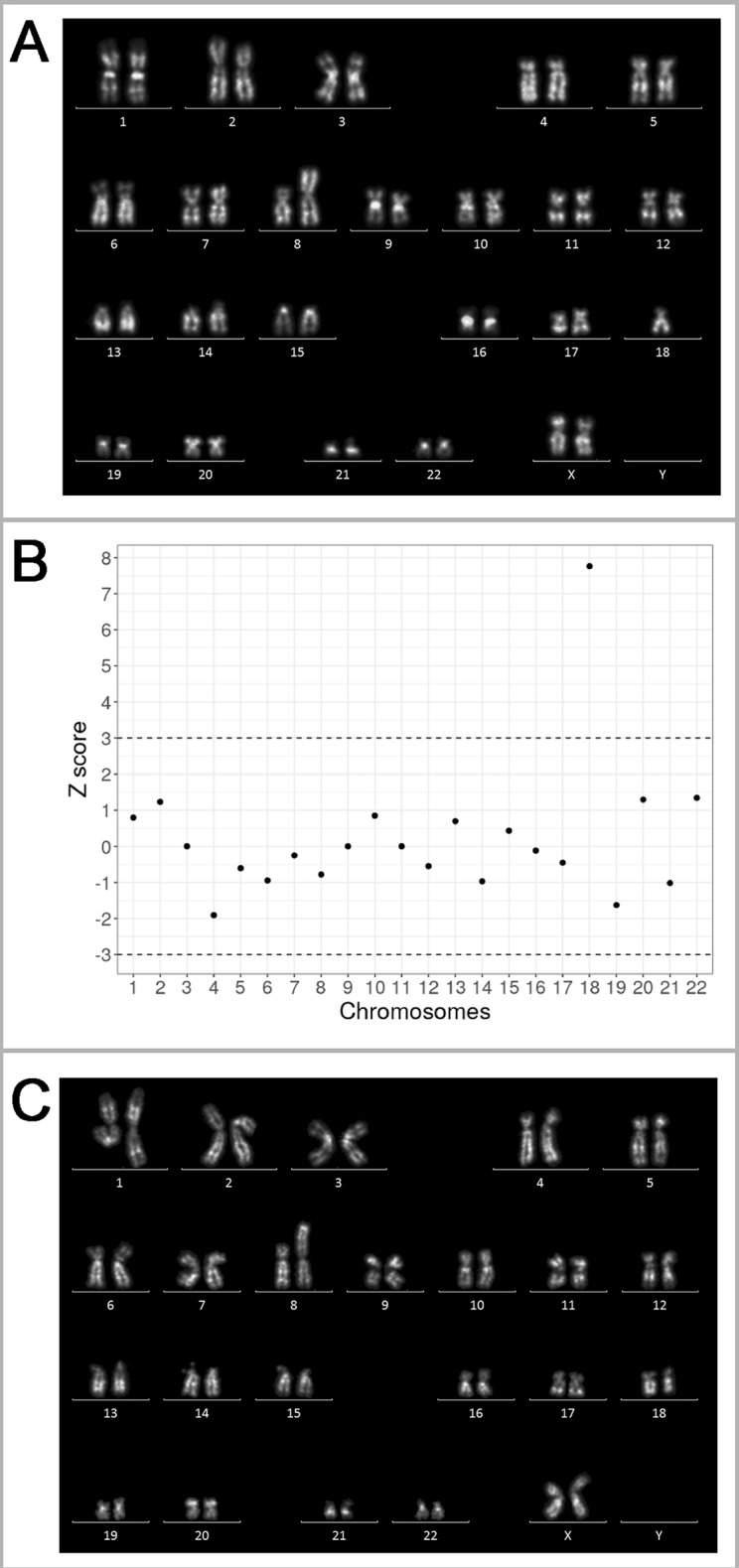
Genetic outcomes of the natural pregnancies in the patient with unbalanced karyotype 45,XX,der(8;18)t(8;18)(p23;p11.3). **(A)** The karyogram of QFH/AcD banded chromosomes from chorionic villi of the miscarriage specimen at 5/6 weeks of gestation, karyotype 45,XX,der(8;18)t(8;18)(p23;p11.3)mat. **(B)** Summary of non-invasive prenatal testing (NIPT) results in progressing pregnancy at 9/10 weeks of gestation. Z scores for the corresponding chromosomes are present on the y-axis. The dash-lines represent the upper and lower limits of the z score for euploidy. The 18 chromosome score is classified as trisomy. **(C)** The karyogram of QFH/AcD banded chromosomes from chorionic villi in progressing pregnancy with trisomy 18 detected by NIPT. The karyotype is 46,ХХ,der(8;18)t(8;18)(p23;p11.3)mat,+18.

The patient experienced three naturally conceived pregnancies: two pregnancies prior to and one after the IVF/PGT-SR cycles. The first naturally conceived pregnancy ended in a miscarriage at 5/6 weeks of gestation. The miscarriage specimen was not karyotyped. The second naturally conceived pregnancy also ended in a miscarriage at 5/6 weeks of gestation. The miscarriage specimen obtained by curettage of the uterine cavity was sent for karyotyping. Chorionic villi were selected and released from the maternal decidua and blood clots under a Leica M125 stereomicroscope. Metaphase chromosomes were prepared from the chorionic villi by the direct technique (without culturing) according to the protocol developed for tissues containing dividing cells (Baranov et al., 1990) with modifications (Pendina et al., 2014; Efimova et al., 2017; Efimova et al., 2018). Karyotyping was performed on QFH/AcD-stained metaphases. An unbalanced karyotype with structurally rearranged chromosome was revealed: 45,ХХ,der(8;18)t(8;18)(p23;p11.3) (**Figure 3A**). The detection of this chromosome abnormality in chorionic villi initiated the story as it was the only indication for karyotyping of the patient and her spouse.

After four unsuccessful IVF/PGT-SR cycles (see *Outcomes of in vitro fertilization protocols*), the patient conceived the third natural pregnancy. NIPT was performed at the gestational age of 9/10 weeks. Blood sample was collected to an EDTA-K2 tube. The plasma was separated by two-step centrifugation. DNA was extracted from 2 ml of plasma using the MagMAX Cell-Free DNA Isolation Kit (Thermo Fisher Scientific Inc., USA). DNA library was prepared using the Ion Plus Fragment Library Kit (Thermo Fisher Scientific Inc.) according to a modified protocol. The library was then templated by an Ion Chef system and sequenced on an Ion Torrent S5 system (Thermo Fisher Scientific Inc.) The sequencing depth of the sample was 0.03×. The reads were aligned to GRCh37 reference and filtered by mapping quality (> 10) and read length (70–130 bp). The reads were divided into 50 kbp bins and Z score was estimated for each bin as described previously (Johansson et al., 2017). According to the previously described protocol (Ivashchenko et al., 2019), the sample was considered to be aneuploid if the average Z score for a chromosome was above 3 or below -3. The NIPT of the patient's sample revealed additional material of chromosome 18 (**Figure 3B**).

To verify the NIPT results by cytogenetic analysis, chorionic villus sampling was performed. The preparation and staining of metaphase chromosomes was made as described above. An abnormal karyotype was detected, as there was only one normal chromosome 8, two normal chromosomes 18, and an aberrant chromosome der(8;18) (**Figure 3C**). Most likely, the fetus inherited the aberrant chromosome der(8;18) together with one normal chromosome 18 from the patient; one more normal chromosome 18 came from the patient's spouse thus forming a karyotype with in fact three copies of chromosome 18. The pregnancy was terminated for medical reasons.

## Discussion

A genotype–phenotype correlation is of high importance for genetic counseling of unbalanced karyotype carriers. In certain cases, the same genetic imbalance may cause different phenotypes including normal ones and severely pathological ones.

Clinical picture is highly variable in patients with small terminal deletions of 8p. Based on phenotype severity, three groups of 8p deletion carriers may be distinguished. The first group is the most numerous and includes children karyotyped during the first year of life because of facial dysmorphism, neurological symptoms (convulsions) and congenital malformations including heart, genitourinary, diaphragmatic and central nervous system defects (Hutchinson et al., 1992; Wu et al., 1996; Pehlivan et al., 1999; Shimokawa et al., 2004). Patients of the second group are referred to genetic counseling and karyotyping because of severe behavioral disturbances with outbursts of aggressiveness and destructiveness and mild mental defects in childhood (Gardner et al., 2011). In some cases, the clinical manifestation is similar to that of fragile X chromosome syndrome (Wu et al., 1996). The third group is the smallest one; it includes patients who are referred for genetic counseling because of reproductive disorders. These patients are mentally normal and have no malformations or dysmorphia (Pettenati et al., 1992; Reddy, 1999). In some cases, 8p terminal deletions are associated with autism (Chien et al., 2010).

A variability of the clinical picture is also typical for carriers of 8p duplications. In some cases, children who inherit the duplication from a phenotypically normal parent manifest a number of anomalies including minor facial anomalies (a wide nasal arch), moderate mental retardation and autistic behavior (https://decipher.sanger.ac.uk/). A small duplication limited to the 8p22 region is associated with developmental delay (Buysse et al., 2009). Therefore, patients with 8p terminal deletions as well as with 8p duplications have no common behavioral or phenotypic features that can be classified as specific syndromes.

In contrast, deletion of the chromosome 18 short arm is known as the 18p- syndrome (OMIM #146390). Patients with a deletion of almost the entire 18p feature short stature, microcephaly, dysmorphism, round face, mild to moderate mental retardation and behavioral disturbance (de Grouchy, Turleau, 1984). However, if the deletion does not affect the whole arm, the clinical picture may be less pronounced and may include only some or even none of the listed abnormalities.

The patient reported in this study has a 8p22 duplication and terminal deletions both in 8p and 18p. Surprisingly, she has no physical, mental or behavioral abnormalities. The only reason for karyotyping was a structural chromosome rearrangement detected in the patient's miscarriage specimen. Of all the clinical traits associated with an 8p and 18p imbalance, the patient features only a minor dysmorphism that, in fact, does not exceed that of normal physiology—flattened superciliary arches, a wide nasal arch and a fleshy nose tip. The underlying basis for this highly unexpected genotype-phenotype correlation remains obscure. Although rare cases of phenotypically normal patients with 8p or 18p imbalance are described (Pettenati et al., 1992; Reddy, 1999; https://decipher.sanger.ac.uk/; Liehr, 2019), it is almost unbelievable that a combination of both 8p and 18p terminal deletions accompanied by 8p22 duplication may not cause serious clinical manifestations.

The patient is socially normal, and it is her reasonable wish to realize her reproductive rights. However, the genetic counseling regarding this issue is highly challenging. She is likely to have a good reproductive potential, as could be concluded from her normal hormonal status, normal endometrium and her capability to conceive naturally with no specific effort. Her unusual karyotype abnormality and the resulting increased frequency of abnormal gametes, seems to be the only cause of the patient's reproductive failures.

Carriers of structurally rearranged chromosomes are at high risk of having genetically unbalanced offspring (Gardner et al., 2011). However, the theoretically expected ratio of balanced and unbalanced gametes depends on the type of structural rearrangement. In the patient reported in this study, in the case of alternative segregation, the balanced gametes are only those with a normal chromosome 8 and a normal chromosome 18. The counterparts receiving der(8;18) chromosome are unbalanced. All other segregation types form unbalanced gametes. Therefore, the patient theoretically has two times lower chance of balanced gametes than the carriers of reciprocal translocations. Moreover, segregation of dicentric chromosome may result in *de novo* chromosomal rearrangements including complex ones such as chromothripsis (Koltsova et al., 2019). Indeed, among 11 genetically analyzed products of conception (one miscarriage, 9 preimplantation embryos and one prenatally analyzed embryo), only one was balanced regarding the rearrangement. The other embryos were unbalanced; they demonstrated different combinations of normal chromosomes 8 and 18 and the rearranged one with no prevalence of any of the variants.

It should be emphasized that inheritance of the patient's derivative chromosome by her offspring together with normal chromosomes 8 and 18 from the patient's spouse is not desirable. Even though in this case the fetus would have the same karyotype as the patient, the phenotypic effect of the aberration may be unpredictable. Pettenati et al. reported on a family where a brother and a sister inherited a 8p23 deletion from their phenotypically normal father. The boy had mental retardation, behavioral problems, convulsions and hydrocephalus. His sister had no mental problems, but was hyperactive (Pettenati et al., 1992). In another study, a 6-month-old girl who inherited a 8p23.1∼23.2 deletion from her asymptomatic father also had no clinical manifestations (Reddy, 1999).

Considering the very low chance of balanced gametes, the emotional stress caused by numerous unsuccessful attempts to conceive a balanced embryo and the increasing age of the patient, which in turn elevates the risk of other aneuploidies, the patient has been offered an IVF cycle with a donor oocyte. The patient has a strong intention to conceive a karyotypically normal fetus, but she has not yet decided whether to make another attempt of the IVF/PGT-SR cycle with her own oocytes or to use donor cells.

## Data Availability Statement

The datasets generated for this study are available on request to the corresponding author.

## Ethics Statement

The studies involving human participants were reviewed and approved by Ethics Committee of D.O. Ott Research Institute of Obstetrics, Gynecology and Reproductology. The patients/participants provided their written informed consent to participate in this study. Written informed consent was obtained from the individual(s) for the publication of any potentially identifiable images or data included in this article.

## Author Contributions

Design and conception of the study: AP, YVS, OT, OE, IK. Laboratory investigations and diagnostic tests: AP, YVS, OT, OC, OM, VD, OE, LP, AK, AT, TT, NO, MK, AP-K, TK, TI, GT, OG, OR, AS, SU, VT, YE, SS, YMS, IM, EK, EV, PK, ND, ASG, AMG, IK. Genetic counseling: EAS, ESS. Writing of the manuscript: OE, AP. Checking of the manuscript for important intellectual content: OC, VB, AMG, IK. All authors read and approved the submitted version.

## Funding

The study was performed within the framework of the Basic Research Program funded by the Ministry of Science and Higher Education of the Russian Federation in 2019-2021 (program number АААА-А19-119021290033-1). AK and MK are grantees of RF President Scholarship.

## Conflict of Interest

Author ND was employed by company NIPT LLC (St. Petersburg, Russia).

The remaining authors declare that the research was conducted in the absence of any commercial or financial relationships that could be construed as a potential conflict of interest.

## Abbreviations

aCGH, array comparative genomic hybridization; AMH, anti-müllerian hormone; BMI, body mass index; CNV, copy number variation; DHEA, dehydroepiandrosterone; ER, estrogen; FISH, fluorescent *in situ* hybridization; FSH, follicle-stimulating hormone; ICSI, intracytoplasmic sperm injection; IVF, *in vitro* fertilization; GnRH, gonadotropin-releasing hormone; GV, germinal vesicle; LH, luteinizing hormone; NIPT, non-invasive prenatal testing; OMIM, Online Mendelian Inheritance in Man; PGT-SR, preimplantation genetic testing for structural chromosomal aberrations; PHA, phytohemagglutinin; PR, progesteron; QFH/AcD, quinacrine fluorescence Hoechst/actinomycin D.
